# Labelled IoT flow-based network traffic dataset for cyberattack detection

**DOI:** 10.1016/j.dib.2026.112717

**Published:** 2026-03-23

**Authors:** Branly Martínez, Carlos Cambra, Daniel Urda, Jaime Rincón, Álvaro Herrero

**Affiliations:** Grupo de Inteligencia Computacional Aplicada (GICAP), Departamento de Digitalización, Escuela Politécnica Superior, Universidad de Burgos, Av. Cantabria s/n, Burgos 09006, Spain

**Keywords:** Internet of things, Cybersecurity, Flow features, Attack traffic, Benign traffic, Network, Ground truth, Labelling

## Abstract

This data article presents a labelled flow-based network traffic dataset collected from a controlled Internet of Things (IoT) laboratory environment. The dataset captures network communication generated by Raspberry Pi-based IoT nodes configured to emulate service and client roles. Traffic was recorded during normal operations and during the execution of predefined cyberattack scenarios within an isolated experimental network.

Network traffic was recorded at the packet level using passive network monitoring and stored in PCAPNG format. The packet captures were subsequently processed into bidirectional network flows, producing flow records with statistical and temporal attributes derived from the observed packet exchanges.

Cyberattack-related flows were labelled using the experimental ground-truth markers recorded during each attack campaign, complemented by the fixed attacker node IP address. Flows outside the marked intervals were labelled as benign and corresponded to regular device communication. This combined labelling approach reduces the potential for overlap between benign and attack activities.

The dataset covers nine attack scenarios grouped into six attack categories. It is released through a structured repository containing raw packet captures, labelled flow files, and supporting metadata for flow-based IoT traffic analysis, cyberattack detection research, and optional re-labelling.

## Graphical abstract

**Figure:**
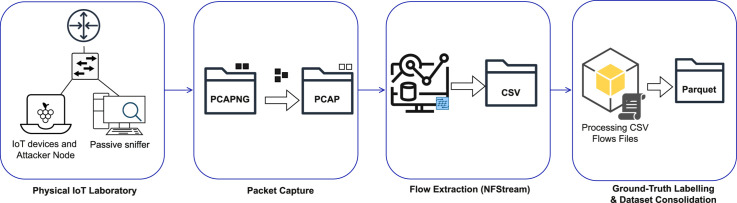

Specifications Table

**Table:** 

Subject	Computer Sciences
Specific subject area	Flow-based IoT network traffic dataset for cyberattack detection.
Type of data	Raw network packets (PCAPNG/PCAP); Processed network flows (CSV/PARQUET); Experimental metadata (TEXT, JSON)
Data collection	Network traffic was generated in a controlled physical IoT testbed composed of multiple Raspberry Pi–based nodes assigned fixed roles, including service, client, traffic-generation, and attacker functions. Devices were interconnected through a managed Ethernet switch with port mirroring enabled. Network traffic was passively captured using Tshark and stored in PCAPNG format during normal operation and predefined cyberattack executions. The captured packet traces were subsequently processed into bidirectional network flows, and attack-related flows were identified based on documented attack execution time intervals.
Data source location	The data were collected in: Universidad de Burgos (Escuela Politécnica Superior, Campus Río Vena) City/Town/Region: Burgos, Burgos, Castilla y León Country: Spain Geographical coordinates: 42.351000, −3.689000
Data accessibility	Repository name: RiUBU (Universidad de Burgos Institutional Repository) / SCAYLE (file storage) Data identification number: https://doi.org/10.71486/dyxt-2r24 [1] Direct URL to data: https://doi.org/10.71486/dyxt-2r24 Instructions for accessing these data: The DOI resolves to the RiUBU landing page (handle record), which contains the dataset metadata and a direct download link hosted on SCAYLE.
Related research article	None.

## 1 Value of the Data

• The dataset provides labelled flow-level network traffic collected under controlled and documented experimental conditions, supporting reproducible evaluation of cyberattack detection approaches.
• The data can be reused by researchers to develop, test, and compare flow-based cyberattack detection approaches for IoT networks, including supervised learning methods and statistical traffic analysis techniques.
• The inclusion of raw packet captures, processed flow-level data, and experimental metadata enables users to inspect the processing chain, revise the released labels if required, and derive alternative feature sets for specific research needs.
• The dataset can support comparative studies involving IoT network traffic characterization and the evaluation of flow-based cyberattack detection methods, as well as educational use in experimental cybersecurity and IoT networking courses.

## 2 Background

The increasing deployment of IoT systems in industrial and operational environments has led to growing interest in network-based approaches for identifying malicious activities affecting connected devices. In this context, flow-based network traffic analysis has become a commonly used method due to its scalability and suitability for resource-constrained environments.

Several public IoT network traffic datasets have been proposed in recent years. Bot-IoT [2] provides traffic collected to support IoT botnet and forensic analytics, while N-BaIoT [3] focuses on network-based botnet detection for consumer IoT devices. IoT-23 [4] offers labelled malicious and benign IoT traffic captures, and CICIoT2023 [5] provides a recent multi-protocol IoT dataset intended for intrusion detection research. In addition, Edge-IIoTset [6] targets both IoT and IIoT scenarios and has been used in centralized and federated learning evaluations.

However, as highlighted by surveys of intrusion detection datasets [7] and evaluation architectures such as TON_IoT [8], many existing datasets present recurring limitations. These include reliance on simulated environments, incomplete experimental documentation, and limited traceability between attack executions and observed traffic. These gaps motivate the generation of datasets collected under controlled laboratory conditions, where device behavior, network topology, and attack timelines can be explicitly defined and documented.

The dataset presented in this article was generated to provide flow-level network traffic collected under documented laboratory conditions, with explicit correspondence between experimental activities and released data artifacts.

## 3 Data Description

Table 1 summarizes the overall class distribution of the released flow dataset and reports the total number of benign and attack flows.

**Table 1:** Class distribution of the released flow dataset (flow-level).

Class	# Flows	Percentage
Benign	2,875,481	79.49%
Attack	741,907	20.51%
Total	**3,617,388**	**100.00%**

To further characterize the malicious portion of the dataset, Table 2 provides the distribution of attack flows by attack traffic type.

**Table 2:** Attack-flow distribution by attack traffic type.

Attack type	# Attack flows	% of attack flows	% of total flows
Denial of Service	593,576	80.01%	16.41%
ARP Spoofing (MITM)	61,042	8.23%	1.69%
Nmap Reconnaissance	51,155	6.90%	1.41%
SQL Injection	33,814	4.56%	0.93%
DNS Beaconing	1,611	0.22%	0.04%
MQTT Injection	486	0.07%	0.01%
Brute Force	223	0.03%	0.01%
Total attack	741,907	100.00%	20.51%

*Denial of service* traffic represented the largest share of attack flows, followed by *ARP spoofing* (MITM), *reconnaissance scanning*, and *SQL injection*.

In addition to the class and attack-type distributions, the released data are provided through a hierarchical repository containing the main artifacts associated with this work.

### 3.1 Repository structure

• **01_Raw:** Original PCAPNG packet capture files produced during the experimental campaigns.
• **02_Pcap:** PCAP versions of the original packet captures, provided for compatibility with downstream processing.
• **03_Flows:** Unlabelled flow-level CSV files extracted from packet captures, with one file per capture session.
• **ATK_Metadata:** JSON metadata files describing each attack execution, including identifiers, attack type, timestamps, duration, and related contextual parameters.
• **04_Manifests:** Mapping files linking packet captures, attack identifiers, and temporal windows used during labelling and dataset consolidation.
• **05_Labeled_Flows:** Labelled flow-level files that retain the extracted features and include the ground-truth variables used in the released dataset, such as traffic class and attack type.
• **06_Datasets:** Consolidated dataset files stored in Parquet format, along with a summary CSV file for inspection.

### 3.2 Description of dataset variables

The final dataset is presented in a flow-level tabular format. The variables describe flow identification, temporal dynamics, traffic volume, protocol characteristics, application-level metadata, and ground-truth labelling information.

Table 3 organizes the dataset variables into functional categories and lists the representative attributes for each group.

**Table 3:** Description of key variables in the processed dataset.

Feature category	Description	Representative attributes
Flow identifiers	Attributes that uniquely identify a network flow and its endpoints.	src_ip, dst_ip, src_port, dst_port, protocol, ip_version, id
Temporal information	Variables describing flow timing, duration, and observation windows.	bidirectional_duration_ms, bidirectional_first_seen_ms, bidirectional_last_seen_ms, t_start_expanded_ms, t_end_expanded_ms
Packet statistics (bidirectional)	Packet-level counts and statistics aggregated over the entire bidirectional flow.	bidirectional_packets, bidirectional_ack_packets, bidirectional_syn_packets, bidirectional_fin_packets
Packet statistics (directional)	Packet counts and statistics computed separately for each traffic direction.	src2dst_packets, dst2src_packets, src2dst_syn_packets, dst2src_rst_packets
Byte and volume statistics	Features describing the volume of data exchanged within the flow.	bidirectional_bytes, src2dst_bytes, dst2src_bytes
Inter-arrival time and packet size statistics	Statistical descriptors of packet inter-arrival times and packet sizes.	bidirectional_mean_piat_ms, bidirectional_stddev_piat_ms, src2dst_max_ps, dst2src_min_ps
TCP flags and header indicators	Indicators derived from TCP header flags observed during the flow.	syn_packets, ack_packets, fin_packets, rst_packets, psh_packets, urg_packets
Application-level metadata	Attributes related to application identification and protocol inspection.	application_name, application_category_name, application_confidence, requested_server_name, user_agent
Fingerprinting and protocol hints	Metadata derived from client and server fingerprinting techniques.	client_fingerprint, server_fingerprint, content_type
Network layer metadata	Attributes describing network-level context and encapsulation.	vlan_id, tunnel_id, window_mode
Ground-truth and labelling	Variables associated with flow labelling and attack traceability.	label, attack_type, traffic_kind, gt_start_ms, gt_end_ms
Provenance and traceability	Metadata enabling reproducibility and linkage to original data sources.	pcap_path, pcap_sha256, source_pcap, source_csv, expiration_id

## 4 Experimental Design, Materials and Methods

This section summarizes the experimental setup, traffic generation process, network capture configuration, and flow extraction and labelling workflow applied to the raw packet traces.

### 4.1 Experimental setup and network environment

The dataset was generated in a controlled physical laboratory environment designed to model a small-scale Internet of Things (IoT) network. The experimental testbed consisted of multiple interconnected nodes deployed in a private local area network. Each node was assigned a fixed and well-defined role throughout all experimental campaigns.

IoT endpoints were implemented using Raspberry Pi devices configured as service and client nodes, thereby enabling the bidirectional communications characteristic of the experimental IoT setup. The infrastructure included a dedicated attacker host, an IoT data hub hosting an MQTT broker and application-level forwarding services, and a SCADA-like server providing web-based services and database storage. A separate traffic-generation node emulated multiple virtual IoT devices through IP aliasing. This configuration enabled the generation of concurrent network flows from multiple logical sources.

All Raspberry Pi nodes ran Debian GNU/Linux 13 (Trixie, version 13.3). The IoT data hub node hosted Eclipse Mosquitto version 2.0.21 as the MQTT broker. The SCADA-like server node ran Apache HTTP Server version 2.4.66 and MariaDB version 11.8.3, to provide web-based monitoring and database storage services. Traffic traces were handled on a dedicated monitoring workstation running Windows 11 (25H2) using TShark (Wireshark) version 4.6.4.

**Fig. 1:**
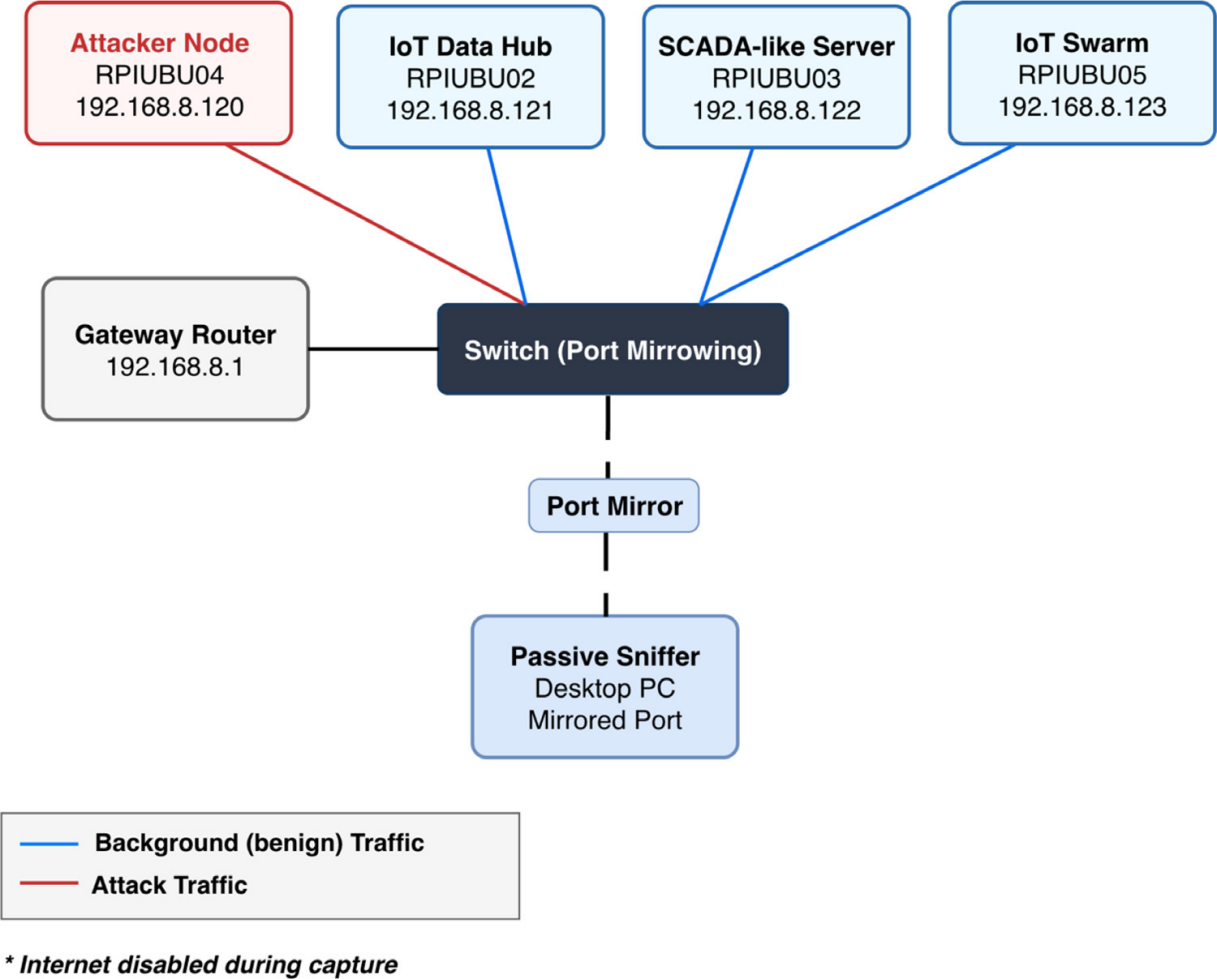
Physical testbed topology, node roles, and passive port-mirroring capture setup.

Interconnections between the nodes were achieved using a managed Ethernet switch configured with port mirroring. Fig. 1 summarizes the physical testbed topology and the passive acquisition setup used throughout all the campaigns.

All devices used static IPv4 addressing within a private address space to ensure deterministic communication patterns and reproducibility. The laboratory network was fully isolated from external or production environments, and Internet connectivity was disabled during data collection. Consequently, all captured traffic corresponded exclusively to benign operational behavior or intentionally generated experimental activities.

Network communications were conducted over the TCP/IP within a wired Ethernet local area network. The network topology, device roles, addressing scheme, and capture configuration were kept constant across all experimental sessions to ensure comparability and repeatability of the collected data.

### 4.2 Traffic generation scenarios

Network traffic was generated under controlled laboratory conditions to capture both benign operational behavior and intentionally executed experimental events representative of the IoT testbed.

Benign traffic was produced continuously throughout the experiments to establish a baseline for normal network behavior. This traffic comprises periodic telemetry exchanges, service requests, and background communications typical of IoT device operation.

In addition to benign activity, predefined experimental events were generated during specific time intervals. For each attack campaign, a packet capture was started when benign traffic was running, the corresponding event was executed within the defined interval, and the capture was stopped and saved as an attack-specific trace. The exact start and end timestamps of each event were recorded for subsequent flow-level labelling.

Table 4 summarizes the cyberattack traffic generation scenarios included in the dataset and indicates the attack category, type, tools employed, and targeted services or protocols.

**Table 4:** Summary of cyberattack traffic generation scenarios included in the dataset.

Attack category	Attack type	Tool used	Target service / protocol	Description
Man-in-the-Middle	ARP spoofing (MITM)	arpspoof	ARP / Ethernet	Address Resolution Protocol spoofing activities performed to place the attacker between communicating IoT devices, enabling passive interception and relay of network traffic during predefined time intervals.
Denial of Service	ICMP flooding	hping3	ICMP / Network layer	High-rate ICMP echo request packets generated to overload network resources and induce abnormal traffic patterns at the network layer.
Denial of Service	TCP SYN flooding	hping3	TCP-based services (e.g., HTTP, MQTT)	High-rate TCP SYN packet generation targeting application services to produce connection handling anomalies and resource exhaustion conditions.
Denial of Service	MQTT flooding	Custom script	MQTT (TCP)	High-frequency MQTT PUBLISH message generation targeting the broker to generate abnormal traffic volumes and protocol-level stress conditions.
Beaconing activity	DNS beaconing	Custom script	DNS (UDP)	Periodic DNS queries generated to simulate beaconing behavior commonly associated with command-and-control communication patterns.
Credential access	Brute-force authentication	Hydra	SSH (TCP)	Automated dictionary-based authentication attempts directed at the SCADA server during predefined attack intervals.
Injection	MQTT message injection	Custom script	MQTT (TCP)	Malicious MQTT messages injected into the broker to simulate unauthorized data manipulation within IoT communication channels.
Injection	SQL injection	Sqlmap	HTTP / Database backend	Automated HTTP requests generated to test SQL injection patterns against the web-based monitoring dashboard and backend database services.
Reconnaissance	Network and port scanning	Nmap	TCP/IP	Active network and port scanning activities performed to enumerate hosts and exposed services within the IoT laboratory environment.

### 4.3 Network traffic capture

Network traffic was passively captured during all experimental sessions using a dedicated monitoring host connected to a mirrored port on the managed Ethernet switch. Traffic capture was performed continuously throughout each experimental session. Packet traces were recorded at the network level and stored in PCAPNG format to preserve timestamps and protocol headers.

The monitoring host was exclusively used for traffic acquisition and did not participate in traffic generation or experimental activities. Captured packet traces were organized and stored according to experimental sessions, preserving the direct correspondence between capture files and the associated traffic generation scenarios.

### 4.4 Flow extraction and feature generation

Following packet capture, the raw network traffic traces were processed into flow-level representations. Packet capture files stored in PCAPNG format were first converted to PCAP to ensure compatibility with NFStream.

Flow extraction was performed using NFStream (v6.5.4) [9], which aggregates packet-level information into bidirectional network flows. The extraction configuration used an active timeout of 60 s (active_timeout = 60) and an idle timeout of 15 s (idle_timeout = 15). Built-in dissections were enabled by inspecting up to 20 packets per flow (n_dissections = 20) to extract application-level metadata fields (e.g., application_name, user_agent) without additional plugins. Each bidirectional flow represents a communication instance between two network endpoints and is characterized by temporal, statistical, and protocol-related features derived from the underlying packet sequence.

These values were selected as fixed flow-extraction parameters for dataset generation. The active timeout of 60 s, which is shorter than the NFStream default, limited the persistence of long-lived communications and produced shorter flow records for downstream labelling and comparison. The idle timeout of 15 s allowed flows to expire after short communication pauses, reducing the aggregation of temporally separated exchanges into a single record. The dissection depth of 20 packets, corresponding to the NFStream default, provided a bounded and uniform inspection depth for application-level metadata extraction across all captures.

The same extraction configuration and feature schema were applied to all packet captures. The resulting flow records were exported in tabular format, with each row corresponding to a bidirectional flow and each column representing a specific feature. These unlabelled flow files were subsequently used for labelling and dataset consolidation.

### 4.5 Ground-truth labelling procedure

Ground-truth labels were assigned to the extracted flow-level data using a reproducible labelling procedure based on temporal correlation with documented experimental activities. Labelling was performed after flow extraction to ensure that packet-level processing and feature generation remained independent of the annotation process.

Each experimental activity was associated with explicit start and end timestamps recorded during execution. These timestamps were stored as structured metadata and used to define labelling windows. A flow was considered associated with an activity if its temporal boundaries overlapped with the corresponding time window. In the released dataset, each attack capture session contained a single labelled event window associated with one experimental activity. Therefore, the dataset does not include cases in which a flow overlaps multiple attack windows from distinct attack events.

Flows whose temporal extent partially overlapped with the event-window boundaries were still considered candidates for labelling, subject to scenario-specific labelling criteria. In addition, the NFStream timeout settings limited the persistence of long-lived connections by segmenting them into shorter flow records.

For ARP spoofing (MITM), flows were labelled based solely on temporal overlap with the recorded event window because the attacker relays traffic between victim nodes and may therefore not appear as a flow endpoint. For the remaining attack campaigns, labels were assigned using temporal overlap together with the fixed attacker IP address. This auxiliary constraint reduced the attribution of concurrent benign traffic within the same capture window. Based on the released dataset composition, this temporal-and-IP rule accounts for 680,865 of the 741,907 labelled attack flows (91.77%), whereas 61,042 attack flows (8.23%) corresponding to ARP spoofing (MITM), were labelled by temporal overlap alone.

Labelling information was stored as dedicated variables within the flow-level datasets, including a binary traffic class label and a categorical descriptor indicating the type of experimental activity. Auxiliary metadata files were preserved separately to maintain traceability between flow records, packet captures, and original experimental execution logs.

### 4.6 Dataset validation and quality control

The released labels relied on a centralized capture workflow using a dedicated monitoring host connected to the mirrored switch port, along with recorded event windows stored as structured metadata. Thus, label assignment did not depend on combining independently timestamped packet traces from multiple nodes. As part of the processing pipeline, capture files, temporal metadata, and generated flow files were cross-referenced using the mapping files provided in the 04_Manifests folder to preserve traceability and detect inconsistencies before dataset consolidation.

In addition, the consolidated dataset (3,617,388 flows) was subjected to post-hoc sanity checks. All 680,865 non-MITM attack flows were confirmed to contain the fixed attacker IP address as either source or destination endpoint, with zero exceptions. No attack-labelled flow was found outside its corresponding event window. The resulting attack-type distribution was also reviewed to confirm consistency with the expected composition of the released dataset. The correspondence between capture files, temporal metadata, and generated flow records was verified throughout the dataset consolidation.

## Limitations

The dataset was collected in a laboratory testbed and may not fully reflect the diversity, scale, and variability of real-world IoT deployments. Consequently, the observed traffic patterns may differ from those present in operational networks comprising heterogeneous devices and dynamic usage conditions. All captures were collected over a fixed wired LAN with passive port mirroring and no Internet access.

The scope of the dataset is limited to the experimental activities executed during the data collection campaigns. Although multiple categories of network activity were included, the dataset does not cover the full range of possible IoT communication behaviors or anomalous events that may arise in uncontrolled environments. Accordingly, the released data cover the nine scenarios summarized in Table 4 and should not be interpreted as exhaustive.

Ground-truth labelling relies on temporal correlation with documented experimental activities, supported by per-campaign markers and a fixed attacker IP address, to minimize ambiguity at event boundaries. Nevertheless, this approach assumes controlled execution conditions and access to accurate timing and marker information, which may not be available in all data collection contexts.

Finally, the dataset represents the network behavior under fixed configurations and roles. Variations in device firmware, network configurations, or protocol implementations were not explored within the scope of this data collection. Future releases may extend this baseline by varying the devices, configurations, and deployment conditions.

## Ethics Statement

The authors have read and followed the ethical requirements for publication in Data in Brief and have confirmed that the present work does not involve human subjects, animal experiments, or any data collected from social media platforms.

## CRediT Author Statement

**Branly Martínez:** Conceptualization, Methodology, Software, Investigation, Data Curation, Validation, Formal analysis, Visualization, Writing – Original Draft, Writing – Review & Editing; **Carlos Cambra:** Supervision, Writing – Review & Editing; **Daniel Urda:** Supervision, Writing – Review & Editing; **Jaime Rincón:** Supervision, Writing – Review & Editing; **Álvaro Herrero:** Project administration, Funding acquisition.

## Data Availability

RiUBU/SCAYLECyberFlowIoT-GICAP: Labelled Flow-Based Network Traffic Dataset for Cyberattack Detection (Original data).
